# A Network-Based Bioinformatics Approach to Identify Molecular Biomarkers for Type 2 Diabetes that Are Linked to the Progression of Neurological Diseases

**DOI:** 10.3390/ijerph17031035

**Published:** 2020-02-06

**Authors:** Md Habibur Rahman, Silong Peng, Xiyuan Hu, Chen Chen, Md Rezanur Rahman, Shahadat Uddin, Julian M.W. Quinn, Mohammad Ali Moni

**Affiliations:** 1 Institute of Automation, Chinese Academy of Sciences, Beijing 100190, China; habib@ia.ac.cn (M.H.R.); silong.peng@ia.ac.cn (S.P.); xiyuan.hu@ia.ac.cn (X.H.); chen.chen@ia.ac.cn (C.C.); 2 University of Chinese Academy of Sciences, Beijing 100190, China; 3 Department of Computer Science and Engineering, Islamic University, Kushtia 7003, Bangladesh; 4 Department of Biochemistry and Biotechnology, Khwaja Yunus Ali University, Enayetpur, Sirajgonj 6751, Bangladesh; rezanur12@yahoo.com; 5 Complex Systems Research Group & Project Management Program, Faculty of Engineering, The University of Sydney, Sydney, NSW 2006, Australia; shahadat.uddin@sydney.edu.au; 6 Bone Biology Division, Garvan Institute of Medical Research, Darlinghurst, NSW 2010, Australia; j.quinn@garvan.org.au; 7 School of Medical Sciences, Faculty of Medicine and Health, The University of Sydney, Sydney, NSW 2006, Australia

**Keywords:** bioinformatics, computational biology, gene ontology, protein, pathways, type 2 diabetes, neurological disease

## Abstract

Neurological diseases (NDs) are progressive disorders, the progression of which can be significantly affected by a range of common diseases that present as comorbidities. Clinical studies, including epidemiological and neuropathological analyses, indicate that patients with type 2 diabetes (T2D) have worse progression of NDs, suggesting pathogenic links between NDs and T2D. However, finding causal or predisposing factors that link T2D and NDs remains challenging. To address these problems, we developed a high-throughput network-based quantitative pipeline using agnostic approaches to identify genes expressed abnormally in both T2D and NDs, to identify some of the shared molecular pathways that may underpin T2D and ND interaction. We employed gene expression transcriptomic datasets from control and disease-affected individuals and identified differentially expressed genes (DEGs) in tissues of patients with T2D and ND when compared to unaffected control individuals. One hundred and ninety seven DEGs (99 up-regulated and 98 down-regulated in affected individuals) that were common to both the T2D and the ND datasets were identified. Functional annotation of these identified DEGs revealed the involvement of significant cell signaling associated molecular pathways. The overlapping DEGs (i.e., seen in both T2D and ND datasets) were then used to extract the most significant GO terms. We performed validation of these results with gold benchmark databases and literature searching, which identified which genes and pathways had been previously linked to NDs or T2D and which are novel. Hub proteins in the pathways were identified (including DNM2, DNM1, MYH14, PACSIN2, TFRC, PDE4D, ENTPD1, PLK4, CDC20B, and CDC14A) using protein-protein interaction analysis which have not previously been described as playing a role in these diseases. To reveal the transcriptional and post-transcriptional regulators of the DEGs we used transcription factor (TF) interactions analysis and DEG-microRNAs (miRNAs) interaction analysis, respectively. We thus identified the following TFs as important in driving expression of our T2D/ND common genes: FOXC1, GATA2, FOXL1, YY1, E2F1, NFIC, NFYA, USF2, HINFP, MEF2A, SRF, NFKB1, USF2, HINFP, MEF2A, SRF, NFKB1, PDE4D, CREB1, SP1, HOXA5, SREBF1, TFAP2A, STAT3, POU2F2, TP53, PPARG, and JUN. MicroRNAs that affect expression of these genes include mir-335-5p, mir-16-5p, mir-93-5p, mir-17-5p, mir-124-3p. Thus, our transcriptomic data analysis identifies novel potential links between NDs and T2D pathologies that may underlie comorbidity interactions, links that may include potential targets for therapeutic intervention. In sum, our neighborhood-based benchmarking and multilayer network topology methods identified novel putative biomarkers that indicate how type 2 diabetes (T2D) and these neurological diseases interact and pathways that, in the future, may be targeted for treatment.

## 1. Introduction

Type 2 diabetes (T2D) is a global health burden that affects hundreds of millions of people [1]. It is characterized by glucose dyshomeostasis, hyperglycaemia and insulin resistance, with predisposing factors that include obesity, poor quality diet, insufficient physical activity and genetic factors [2,3]. These factors interact to cause failure of circulating glucose level regulation which can result in an inability to supply sufficient insulin and eventual beta-cell loss that exacerbates the condition [4,5]. Glucotoxicity caused by chronic hyperglycemia induces cell injury of many cell types but hepatocytes and pancreatic cells in particular [6]. Hyperglycaemia classically causes vascular disease, damaging blood vessels and leading to a range of cardiovascular diseases. In addition, hyperglycemia has a number of long term effects that exacerbate impairments of central nervous system (CNS) function and cognitive function [7]. Metabolic changes seen in T2D patients lead to chronic CNS inflammation that contribute to neurodegeneration [8]. Other T2D associated metabolic disturbances are associated with atrophy in several regions of the brain (e.g., hippocampal) that in turn are associated with cognitive impairment [9]. The brain is a very insulin-sensitive organ, so insulin resistance itself can affect memory and learning [10]. Indeed, glucose levels affect neuronal maintenance, neurogenesis, neurotransmitter regulation, cell survival and synaptic plasticity [11]. Moreover, it is notable that neurodegenerative diseases are accompanied by high production of inflammatory mediators, oxidative stress, Deoxyribonuclic acid (DNA) damage, and mitochondrial dysfunction which in turn also contribute to the degenerative cascade and exacerbate insulin resistance [12]. T2D is also associated with excessive immune system activation [13].

While the detailed mechanisms remains unclear, epidemiological, cognitive, and neuropathological evidence shows associations between T2D and neurodegenerative diseases such as Alzheimer’s disease (AD) [14], amyotrophic lateral sclerosis (ALS) [15], cerebral palsy (CP) [16], epilepsy disease (ED) [17], Huntington’s disease (HD) [18], multiple sclerosis (MS) [19], and Parkinson’s disease (PD) [20]. Interestingly, neuroimaging studies of the CNS of individuals with T2D also show structural alterations that resemble those in neurological disease patients [21]. The high incidence of T2D thus raises issues around its interaction with other diseases occurring in the same individuals.

Epidemiological studies show a particularly strong association between T2D and AD [14]. AD is characterized by the accumulation of β-amyloid (Aβ) into neuritic plaques and the presence of intracellular aggregates of tau protein in neurofibrillary tangles, amylin deposition as well as synaptic loss, neuroinflammation, and neuronal death [14]. Although its etiology still remains unclear, genetic predisposition and aging are strong risk factors for AD [22]. Moreover, the presence of (Aβ) and tau in the pancreas and insulin-sensitive tissues and their roles in inducing peripheral insulin resistance or disruptions in insulin secretion indicate that this may contribute to the incidence of AD [14].

A wealth of evidence indicates a link between T2D and ALS [15]. ALS is a disorder characterized by progressive muscular atrophy, cognitive impairment, and pyramidal deficit, due to the degeneration of upper and lower motor neurons [23]. Motor neuron loss is associated with mutations in the Cu/Zn superoxide dismutase (SOD1) gene in this disease. ALS patients have altered lipid and glucose metabolism with increased energy consumption [24] and hypermetabolism [25]. Nevertheless, the biological mechanisms linking T2D to ALS are yet unclear even though there are clearly important risk factors in common, including environmental factors, higher body mass index (BMI), elevated cholesterol level and hyperlipidemia [26].

T2D also shows associations with CP, a neurodevelopmental disorder characterized by permanent movement-related disabilities, evident in early life, due to abnormalities in the brain centres that control movement and balance [16]. There are developmental issues in the fetus that lead to CP may have a link to maternal T2D incidence [27], and as maternal obesity and T2D might raise the risk of CP occurring [16]. Notably, children with CP may develop T2D as an adult. The etiology of CP is unclear but the role of perinatal factors such as chorioamnionitis hypoxic-ischemic encephalopathy as well as brain injury occurring during the perinatal and postnatal periods may contribute to CP [28].

T2D is also linked to ED [17]. This is a group of neurological diseases characterized by epileptic seizures. The precise relationship between T2D and epilepsy remains unclear. It is known that epilepsy or seizures are associated with autoimmune or inflammatory disorders and in the pro-inflammatory processes [29]. Additionally, hyperglycemia may exert adverse effects on the central nervous system which leads to ED [30]. Some known risk factors including degenerative brain disorders and head injuries, stroke and dementia are considered as predisposing factors to ED [31].

HD is a neurodegenerative disorder caused by an expanded CAG repeat in exon 1 of the huntingtin gene (HTT) encoding huntingtin protein and characterized by progressive disturbances of mood and motor function, and by cognitive dysfunction [32]. The pathogenetic mechanisms behind HD include misfolding and aggregation of the huntingtin protein, oxidative stress, impaired mitochondrial metabolism, excitotoxicity in affected brain regions, and impairment of the ubiquitin-proteasome system [32]. Although Huntington’s disease (HD) is primarily considered a rare neurodegenerative disorder, insulin resistance and impaired glucose metabolism contribute to its development [33].

MS is a chronic inflammatory and progressive immune-mediated disease of the central nervous system (CNS), characterized by a selective and coordinated inflammatory destruction of the myelin sheath, with damage to the axon [34]. Inflammation, demyelination, and axonal degeneration are associated with MS. Moreover, insulin resistance may induce inflammatory responses, oxidative stress and could exacerbate cognitive impairments in individuals with MS [19]. Though the links between T2D and risk of MS incidence is unclear, there are common genetic and environmental factors that contribute to the MS [34].

PD is clinically characterized by severe motor symptoms that include postural instability, resting tremor, muscular rigidity, and slowness of movements and pathologically characterized by the preferential loss of dopaminergic neurons [20]. PD features the presence of intracellular inclusions, known as Lewy bodies, rich in fibrillar α-Synuclein (aS), a protein suggested being involved in synaptic vesicle recycling and docking [35]. Several epidemiological studies have demonstrated that PD patients have impaired insulin signaling and insulin resistance, and hyperglycaemia which play a role to suppress dopaminergic neuronal activity and that decreasing dopamine turnover that contributes to the possible progression of PD [36].

In sum, there is good evidence that there are pathologically and clinically significant relationships between T2D and many NDs but the association has not been widely examined. As the etiology of T2D and NDs are quite complex and their risk factors somewhat tend to overlap, their biological basis and the molecular mechanisms that underlie this link are still not well understood. Finding interactions between T2D and NDs is very difficult, but is of great interest in medical endocrinology. T2D and NDs are very complex diseases in terms of their clinical presentations, and because of this they are hard to study by conventional hypothesis-driven endocrinology research, despite the high clinical importance. Moreover, there is still a lack of bioinformatics studies addressing the relationship between T2D and NDs. The aim of this study was to identify such links between T2D and NDs, since understanding the nature of these links could bring important insights into the mechanisms that underlie these diseases. This led us to employ a bioinformatics system pipeline, to analyze gene expression data from studies of disease-affected tissues for clues to the nature of the relationship between T2D and NDs.

Here, we focus on finding ND-associated differentially expressed genes (DEGs), molecular pathways and putative discriminative biomarkers that are common to both T2D and NDs. We subsequently performed the validation of the results with gold benchmark experimentally validated databases that include dbGaP, OMIM and OMIM Expanded, and literature.

## 2. Materials and Methods

### 2.1. Datasets Employed in This Study

We query datasets from the National Center for Biotechnology Information (NCBI) Gene Expression Omnibus (GEO) [37]. Queries for each disease returns a number of datasets but most of them were discarded for having a sample size below our selected cutoff sample size of at least 7, having no two conditions such as control vs case or control vs treated, replicate datasets, having undesirable formatting or irrelevant experimental focus, RNAseq datasets, and datasets from non-human organisms. Here, we used datasets by seeking those that would minimise any bias and noise for this type of analysis. This process identified 8 datasets that are highly relevant to T2D, AD, ALS, CP, ED, HD, MS, and PD are appropriate for our study.

We analyzed different human gene expression datasets with accession numbers GSE23343 [38], GSE28146 [39], GSE833 [40], GSE31243 [41], GSE22779 [42], GSE1751 [43], GSE38010 [44]. and GSE19587 [45], having control and disease affected individuals for our study. The T2D dataset (GSE23343) contained gene expression data obtained from liver biopsies of 10 T2D hyperglycemic patients and 7 normoglycemic controls using an Affymetrix Human Genome U133 Plus 2.0 arrays. The AD dataset (GSE28146) was microarray data (also Affymetrix U133 Plus 2.0 arrays) on RNA from snap-frozen brain tissue where white matter tissue was extracted by laser capture methods to collect only CA1 hippocampal gray matter. The ALS dataset (GSE833) employing Affymetrix HuGeneFL Hu6800 arrays, was a study of postmortem spinal cord gray matter samples from 7 ALS patients and from 4 control. The CP (GSE31243) dataset was generated from 40 Affymetrix human genome U133A 2.0 microarray studies of hamstring muscle samples from 20 controls (taken during tissue reconstruction) and 20 CP patients. The ED dataset (GSE22779) was a gene expression profile of peripheral blood mononuclear cells from 12 healthy control and individuals 4 with epilepsy in a study of in vivo glucocorticoid treatment; only data from blood cells extracted before glucocorticoid treatment were used. The HD dataset (GSE1751) was taken from peripheral blood cells from 14 healthy control and 12 symptomatic HD-affected individuals along with 5 presymptomatic HD patients; this also used U133A arrays. The MS dataset (GSE38010) using U133A array data from 5 MS patient brain lesions (identified histologically) compared with 2 brain tissue samples from control individuals. The PD dataset (GSE19587) was an analysis taken from affected brain areas of 12 postmortem brains of PD patients and 10 control samples of unaffected brain tissue using Affymetrix U133A Plus 2.0 arrays.

### 2.2. Preprocessing and Identification of Differentially Expressed Genes

We acquired gene expression microarray datasets from the National Center for Biotechnology Information (NCBI) Gene Expression Omnibus (GEO). All these datasets were generated by comparing diseased tissue against controls to identify differentially expressed genes (DEGs) associated with their respective pathology. To make uniform the mRNA expression data from different platforms and to avoid the problems of experimental systems, we normalized the gene expression data comprising disease state and control data by using the Z-score transformation ($Z_{ij}$) for each NDs gene expression profile using the following equation:

(1) $$Z_{ij}=\frac{g_{ij}-\bar{X}}{\sigma_{i}}$$

where $\sigma_{i}$ implies standard deviation and $g_{ij}$ indicates gene expression magnitude *i* in sample *j*. Such a transformation allows direct comparisons of gene expression values across samples and diseases. The datasets were normalized using the Robust Multi-Array Average expression measure (version 1.30.1) as implemented in the “affy” package (version 1.56.0) of the Bioconductor platform (version Rx64 3.3.0) in R. We performed the analysis of the microarray data using Linear Models for Microarray Data (Limma) [46]. Unpaired *t*-test statistic was used to identify genes differentially expressed in patients compared to normal samples. Moreover, to determine the statistical significance between groups, a two-way analysis of variance (ANOVA) with the false discovery rate (FDR) test was performed. Based on standard statistical criteria, a threshold of at least 1 log2 fold change (logFC) and *t*-tests giving a *p*-value of 0.01 were chosen. *p*-value < 0.01 and logFC > 1 for up-regulated genes and *p*-value < 0.01 and logFC < −1 for down-regulated genes were used. Genes with significantly different expression were thus selected. Gene symbols and names extracted for each disease. Gene symbol records with null or missing data were discarded for each disease. We identified both unique genes that were both over and under-expressed in NDs and T2D. We then pairwise compared the DEGs from T2D datasets with that of our AD, ALS, CP, ED, HD, MS, and PD datasets to find DEGs common to T2D and the NDs. Genes with the greatest magnitude up and down-regulation were selected from those common to the individual disease and T2D.

We then applied neighborhood benchmarking and topological methods to show the associations between genes and diseases. A gene-disease network in short GDN was built to identify gene-disease connections, in which nodes can be either diseases or genes; such a network is represented as a bipartite graph where T2D is the center of this network using Cytoscape V 3.6.1. [47]. Diseases are associated when sharing at least one significantly dysregulated gene. For gene-disease association, we consider a set of human diseases, denoted by D and a set of human genes, denoted by G to find whether gene g∈G is associated with disease d∈D. Moreover, we consider that if $G_{i}$ and $G_{j}$ is the sets of genes with significantly up and down-regulated that were associated with diseases $D_{i}$ and $D_{j}$, respectively, then the number of shared dysregulated genes $\left(n_{ij}^{g}\right)$ associated with both disease $D_{i}$ and $D_{j}$ is defined as follows [48]:

(2) $$n_{ij}^{g}=N\left(G_{i}\cap G_{j}\right).$$

The co-occurrence is the number of common genes between two diseases in the GDN and common neighbors identified employing Jaccard Coefficient methods [48], where edge predictions score (association score) for the node pair is:

(3) $$E\left(i,j\right)=\frac{N(G_{i}\cap G_{j})}{N(G_{i}\cap G_{j})},$$

where *G* is the set of nodes and *E* is the set of edges. We also applied R software packages comoR [49], and POGO [50] to cross-check disease comorbidity associations.

### 2.3. Identification of Molecular Pathway and Gene Ontology

To obtain further insights into the molecular pathways and gene ontology (GO) of T2D that overlap with AD, ALS, CP, ED, HD, MS, and PD, we performed gene set enrichment analysis to identify pathways and GO of the overlapping DEGs with EnrichR [51]. Pathways are central to organism responses to stimuli, and pathway-based analysis is a recently developed approach to understand how complex diseases may be related to each other through their underlying molecular mechanisms [52]. GO is a conceptual model for the representation of gene functions and their relationship to gene regulation [53]. We considered 7 pathway databases: KEGG [54], Reactome [55], NCI-Nature [56], WiKi [57], BioCarta [58], Panther [59] and HumanCyc pathway database [60] and Gene ontology (GO) domain: Biological Process (BP).

### 2.4. Protein-Protein Interactions Analysis

Protein-protein interaction networks (PPIs) represent the physical contacts between two or more proteins molecules and are essential to all cell processes [61]. We also generated protein-protein interaction (PPI) networks based on the physical interaction of the proteins of DEGs by using information from the STRING database [62] via Network Analyst using the confidence score 900, where proteins are represented by nodes and protein interactions represented by edges. Using topological parameters, for example, a degree greater than 15°, highly interacting proteins were identified from PPI analysis.

### 2.5. Transcription Factors-microRNA Interactions Analysis

We studied the DEGs-Transcription Factors (TFs) and DEGs-microRNAs (miRNAs) to identify the regulatory biomolecules (i.e., TFs and miRNAs) that regulate DEGs of interest at the transcriptional and post-transcriptional level. We utilized the JASPAR database to analyze the DEGs-TFs interaction [63]. We employed miRNA-DEGs interactions from TarBase [64] and miRTarBase [65]. The topological analysis was performed via Network Analyzer in Cytoscape [47] and Network Analyst [66]. The TFs were screened out based on the degree (≥20) from the DEGs-TFs network. The miRNAs were selected based on the degree (≥15) from the DEGs-miRNAs network.

### 2.6. An Overview of the Analytical Approach

**Figure 1:**
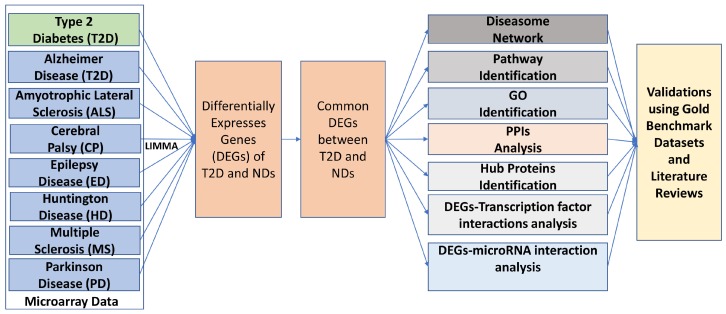
An overview of the network-based systematic and quantitative approach. Neurological diseases that were investigated comprised of Alzheimer’s disease (AD), amyotrophic lateral sclerosis (ALS), cerebral palsy (CP), epilepsy disease (ED), Huntington’s disease (HD), multiple sclerosis (MS), and Parkinson’s disease (PD).

Our network-based systematical and quantitative pipeline to evaluate gene expression in human disease comorbidities is summarized as shown in Figure 1. The integrated pipeline of used here was implemented using the R language, code which is available on request. We developed the proposed pipeline using GEOquery [67] for downloading GEO datasets and expression set class transformation; limma [46] for differentially expressed gene identification from microarray data; genefilter [68] for filtering genes. The version of the used software and packages was R version 3.5.1, R Studio 1.0.136, Bioconductor 3.8, GEOquery 2.50.5, Affy 1.56.0, limma 3.38.3, genefilter 1.64.0.

Our approach employs gene expression analyses, disease gene association networks, signaling pathway mechanisms, gene ontology (GO) data, protein-protein interactions (PPIs) network, DEGs-Transcription Factors (TFs) interaction analysis, and DEGs-MicroRNAs (miRNAs) interaction analysis to identify putative discriminatory biomarkers between T2D and NDs. Furthermore, we also incorporated three gold benchmark verified datasets, OMIM (www.omim.org), OMIM Expanded, and dbGaP (www.ncbi.nlm.nih.gov/gap) to retrieve genes associated with known diseases and relevant disorders for validating the proof of principle of our network-based approach.

## 3. Results

### 3.1. Gene Expression Analysis

To identify and investigate the gene expression effects of T2D that may influence the progression of NDs, we analyzed the gene expression microarray data collected from the National Center for Biotechnology Information (NCBI). Based on significant *p*-values, we found 1320 DEGs for T2D with false discovery rate (FDR) of 0.01 and absolute logFC of 1 using R Bioconductor packages (Limma). Similarly, we identified the most significant DEGs for each ND after statistical analysis. We identified 1606 DEGs in AD, 2901 in ALS, 588 in CP, 1887 in ED, 1338 in HD, 7463 in MS and 1558 in PD which is shown in Table 1.

**Figure 2:**
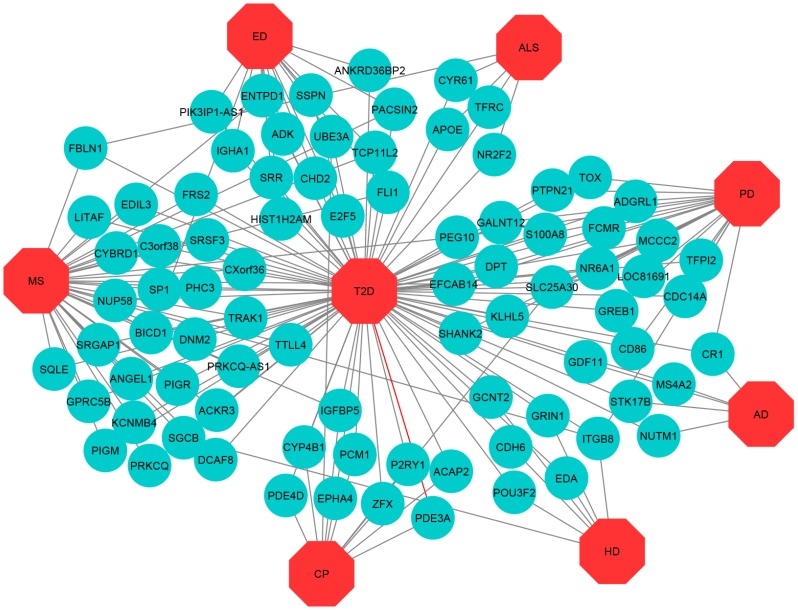
Up-regulated Gene-Disease-network (GDN) between type 2 diabetes (T2D) and neurological diseases (NDs) comprising of commonly up-regulated genes node and different categories of diseases node represented by round shaped robin’s egg blue colour and octagon-shaped red colour.

**Figure 3:**
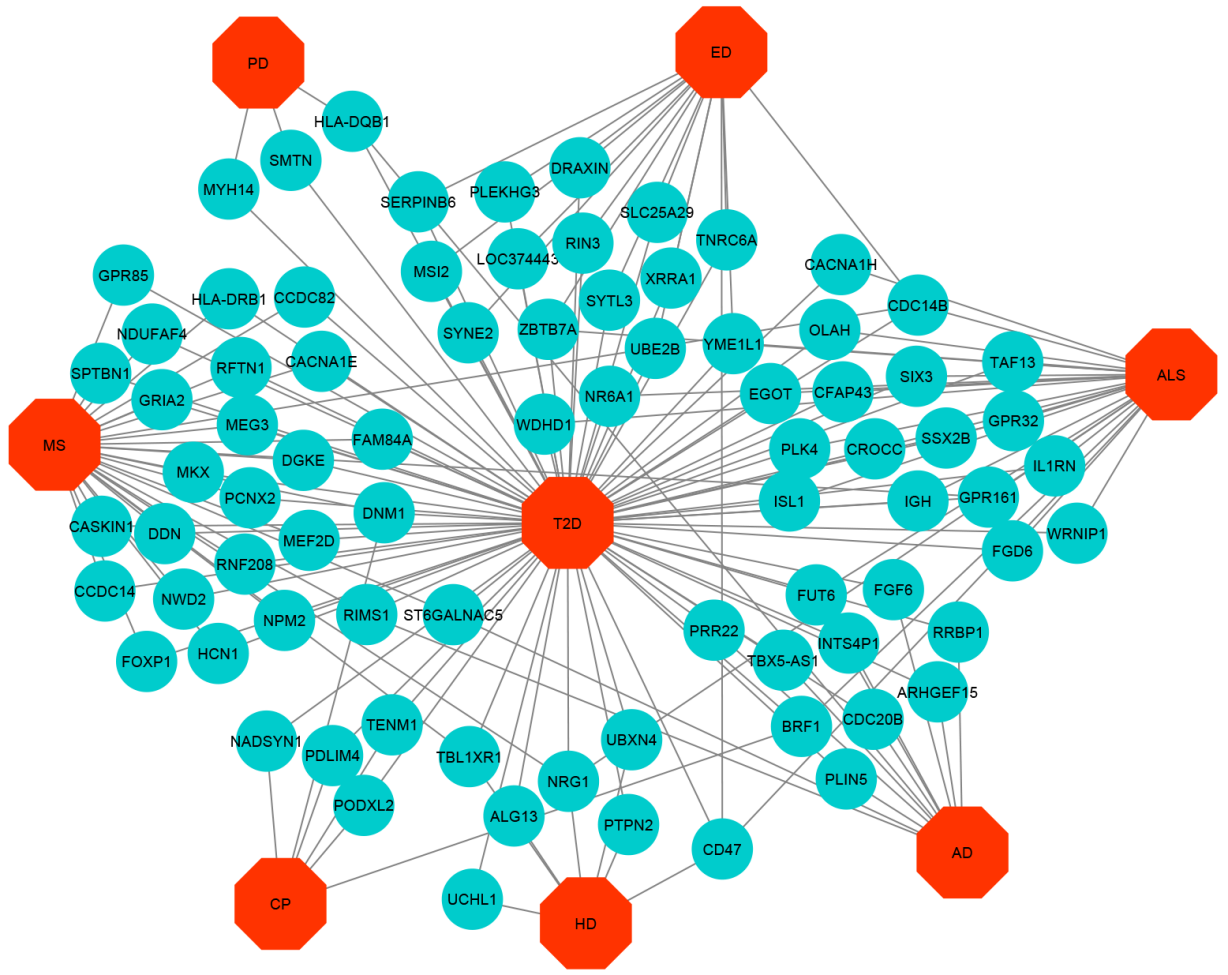
Down-regulated Gene-Disease-network (GDN) between T2D and NDs comprising of commonly down-regulated genes node and different categories of diseases node represented by round shaped robin’s egg blue colour and octagon-shaped red colour.

The cross-comparison analysis also identified common DEGs between T2D and each ND. We found that T2D shares 5, 5, 11, 15, 7, 35 and 21 significantly up-regulated genes whereas 12, 25, 6, 16, 7, 29 and 3 significant down-regulated genes for the AD, ALS, CP, ED, HD, MS, and PD respectively. To get statistically significant associations between T2D and the NDs, we built up- and down-regulated diseasome relationships network centered on the T2D and a link indicated between a disease and a gene when mutations in that gene are known to lead to the specific disease, as shown in Figure 2 and Figure 3 whereas two diseases are comorbid, if they share associated genes.

The most important up-regulated overlapping genes are as follows: (a) FLI1, PACSIN2, BICD1, TCP11L2, and ENTPD1 among T2D, ED, and MS, (b) PEG10 and EFCAB14 among T2D, PD, and MS, (c) ITGB8 among T2D, HD, PD, and MS, (d) FBLN1 among T2D, ALS, and MS, (e) IGFBP5 among T2D, CP, and MS, (f) SGCB among T2D, HD, and MS, (g) SLC25A30 among T2D, PD, and CP. The significant down-regulated overlapping genes are as follows: (a) ST6GALNAC5 and RIMS1 among T2D, AD, and MS, (b) ZBTB7A and YME1L1 among T2D, ALS, and ED, (c) FUT6 among T2D, AD, and ALS, (d) BRF1 among T2D, ALS, and CP, (e) CDC14B among T2D, ALS, ED, and MS, (f) CD47 among T2D, ALS, HD, and ED, (g) NRG1 among T2D, ALS, HD, and MS, (h) DNM1 among T2D, CP, and MS, (i) TLB1XR1 among T2D, HD, and MS, and (j) GPR161 among T2D, ALS, and MS.

### 3.2. Pathway and Functional Association Analysis

We performed pathways analysis to identify how complex diseases are interrelated with other diseases by the underlying molecular mechanisms. We performed gene set enrichment analysis to identify pathways using a bioinformatics resource: EnrichR [51] and considered 7 pathways databases to carry out tests using DEGs common between T2D and each ND. We also performed the regulatory analysis to get more insights into the molecular pathways involved in these comorbidities. We pinpointed overrepresented pathways among DEGs common to T2D and NDs and classified them into functional categories. Pathways deemed significantly enriched in the common DEG sets were reduced by manual curation to include only those pathways which have a *p*-value of below 0.05. We retrieved significant pathways by EnrichR which are significantly connected with DEGs of T2D and NDs as shown in Table 2.

Among the identified pathways, we found that cytokine-cytokine receptor interaction pathway associated with adaptive inflammatory host defenses, cell growth, differentiation, cell death [54]. Glycosphingolipid biosynthesis pathway is associated with abundant amphipathic lipids expression in the nervous system [54]; Ubiquitin proteasome pathway associated with immune response and inflammation, neural and muscular degeneration, morphogenesis of neural networks and response to stress and extracellular modulators [59]; Ionotropic glutamate receptor pathway associated with the mediatation of the majority of excitatory synaptic transmission throughout the central nervous system and synaptic plasticity [59]; Glutamate neurotransmitter release cycle maintains the neurotransmitter glutamate in the central nervous system [55]; Transmission across chemical synapses pathway associated with the communication between neurons, muscle or gland cells [55]; Glutamatergic synapse pathway associated with the regulation of several neuronal functions, such as neuronal migration, excitability, plasticity, long-term potentiation (LTP) and long-term depression (LTD) [57]; Neuronal system pathway comprised of at least 100 billion neurons are associated with the communication among astronomical number of elements with functional connection between neurons [55]; Cell adhesion molecules (CAMs) pathway associated with a vital role in the development and maintenance of the nervous system [54]; Electric transmission across gap junctions pathway associated with the function of communicating neurons in the nervous systems [55]; Transmission across electrical synapses pathway associated with the mechanical conductive link between two neighboring neurons [55]; Spinal cord injury pathway associated with the loss of muscle function, sensation, or autonomic function in the parts of the body [57]; Neurotrophic factor-mediated Trk receptor signaling pathway associated with neuronal differentiation, survival and growth [56]; Neurotrophin signaling pathway associated with differentiation and survival of neural cells [54]; Adipocytokine signaling pathway associated with the process of inflammation, coagulation, fibrinolysis, insulin resistance, diabetes and atherosclerosis [54]; Brain-derived neurotrophic factor (BDNF) signaling pathway associated with growth, differentiation, plasticity, and survival of neurons. BDNF is also implicated in various neuronal disorders such as Alzheimer’s disease, Huntington’s disease [57].

To obtain further insights into the molecular roles and biological significance, enriched common DEGs sets were processed by GO methods using Enrichr, which identifies related biological processes (BP) in order to group them in functional categories. The list of processes and terms was then curated to include those terms with a *p*-value below 0.05. The cell processes thus identified are summarized in Table 3.

### 3.3. Protein-Protein Interactions (PPIs) Analysis

**Figure 4:**
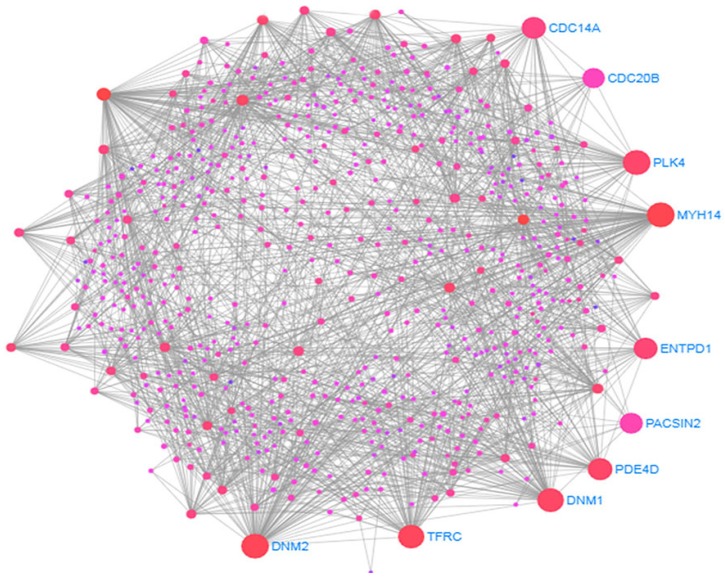
The protein-protein interactions (PPIs) network is built with the significantly dysregulated genes common to type 2 diabetes (T2D) and neurological diseases (NDs).

Using our enriched common disease genesets, we constructed putative PPI networks with web-based visualization resource STRING via Network Analyst using the confidence score 900 by the distinct 159 DEGs, as shown in Figure 4. The PPIs make up the so-called interactomics of the organism where anomalous PPIs cause multiple diseases. Two diseases are known to be related where one or more commonly associated protein subnetworks are shared. Using topological parameters, for example, the degree greater than 15°, highly interacting proteins were identified from PPI analysis.

**Figure 5:**
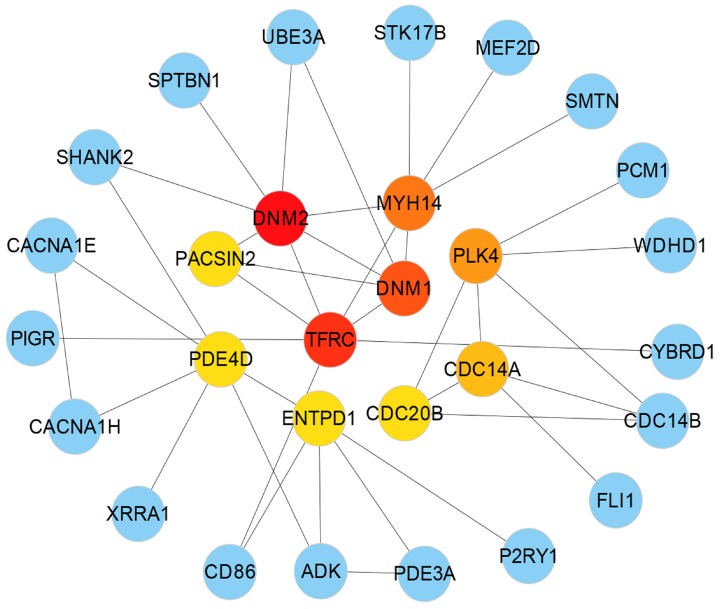
The simplified PPIs network is built with the significantly dysregulated genes common to type 2 diabetes (T2D) and neurological diseases (NDs) to identify the 10 most significant hub proteins marked as red, orange and yellow colour.

The simplified PPI networks were generated with the Cyto-Hubba plugin [69] to identify the most significant hub proteins as shown in Table 4 and the topological parameters were determined by Network Analyzer in Cytoscape [47] as shown in Figure 5.

This data provides evidence that PPI subnetwork exists in our enriched genesets and confirms the inclusion of relevant functional pathways. These identified hub proteins could be useful for therapeutic targets although further characterization is needed for their roles. The summary of hub protein is shown in Table 4.

### 3.4. Identification of Transcriptional and Post-Transcriptional Regulators of the Differentially Expressed Genes

TFs are proteins that regulate transcriptional and gene expression in all living organisms. TFs play a vital role in all cellular processes [70]. miRNAs are short RNA species involved in the post-transcriptional regulation of gene expression. The miRNAs are important biological regulators, for instance, neuronal differentiation, neurogenesis, and synaptic plasticity and they play vital roles in neurodegenerative diseases [71].

**Figure 6:**
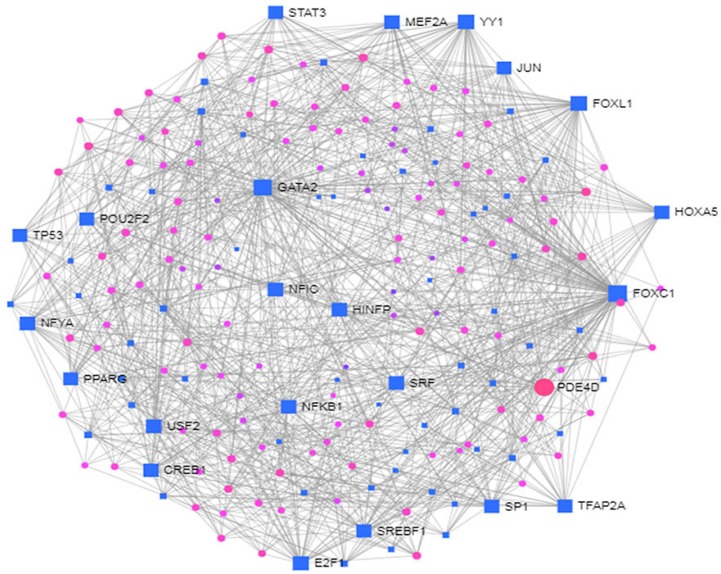
The DEGs-TF interactions regulating the differentially expressed genes common to type 2 diabetes (T2D) and neurological diseases (NDs).

**Figure 7:**
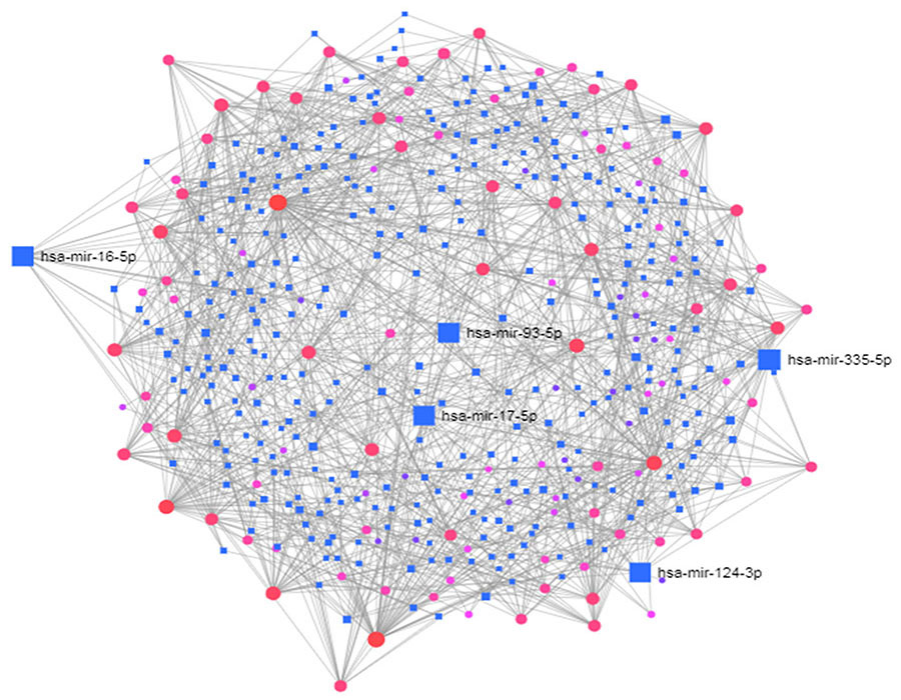
The DEGs-miRNAs interactions regulating the differentially expressed genes common between type 2 diabetes (T2D) and neurological diseases (NDs).

To identify the transcriptional and/or -post-transcriptional regulators of the DEGs, we performed the interaction of the DEGs-TFs analysis as shown in Figure 6 and DEGs-miRNAs interaction analysis as shown in Figure 7.

The biomolecules (i.e., TFs and miRNAs) are summarized in Table 5. The FOXC1, GATA2, FOXL1, YY1, E2F1, NFIC, NFYA, USF2, HINFP, MEF2A, SRF, NFKB1, USF2, HINFP, MEF2A, SRF, NFKB1, PDE4D, CREB1, SP1, HOXA5, SREBF1, TFAP2A, STAT3, POU2F2, TP53, PPARG, JUN were identified as the key regulators of the identified DEGs.

The miRNAs (mir-335-5p, mir-16-5p, mir-93-5p, mir-17-5p, mir-124-3p) were identified to provide an in-depth understanding of the DEGs at post-transcriptional regulators. The summary of transcriptional and/or post-transcriptional regulatory biomolecules of differentially expressed genes that are common to T2D and NDs is shown in Table 5.

### 3.5. Validating Potential Targets Using Gold Benchmark Databases and Literatures

First of all, to validate our identified potential targets, we used OMIM, OMIM Expanded, and dbGaP datasets; these datasets collect curated and validated genes that indicate disease association data from the literature. In the validation, we presented a combined relation of OMIM, OMIM Expanded, and dbGaP databases. For evaluating the validity of our work, we provided statistically significant DEGs (genes common to T2D and neurological diseases (NDs)) to the online tool EnrichR [51] and collected enriched genes and their corresponding neurological disease names from OMIM, OMIM Expanded, and dbGaP databases. To find significant NDs, manual curation is applied considering a *p*-value of 0.05. Then, several diseases such as cancer, infectious diseases are removed from this list because they are not of interest in this study.

**Figure 8:**
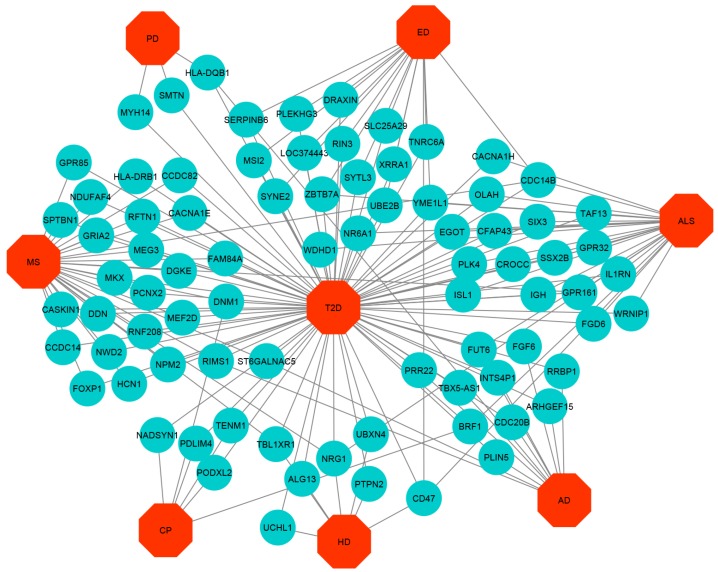
Disease network of type 2 diabetes (T2D) with neurological diseases (NDs) where robin’s egg blue colour round shaped nodes represent genes and octagon shaped red colour nodes represent T2D and NDs.

We also validated our identified potential targets by checking the biomedical literature to find genes clinically used as biomarkers for any of the NDs. We found that Van Cauwenberghe et al. [72] identified the MS4A2 gene associated with AD and Munshi et al. [73] identified the CR1 gene associated with AD. Eykens et al. [74] identified the APOE gene associated with ALS. Fahey et al. [75] identified the TENM1 gene associated with CP. UBE3A and CHD2 genes are associated with ED [76,77]. Arning et al. identified the UCHL1 [78] gene associated with HD. Baranzini et al. [79] identified the HLA-DRB1 gene to be associated with MS. Redenšek et al. identified [80] the HLA-DQB1 gene as associated with PD. This indicates that our analyses of significant genes in NDs match with existing records. We then constructed a Gene-Disease Network (GDN) based on genes and their associated neurological diseases from gold benchmark databases and literature using Cytoscape. This network showed gene-disease associations whereby if a gene mutation is known to lead to a specific disease, a link is indicated between disease and gene; this is shown in Figure 8.

## 4. Discussion

T2D and NDs are complex diseases but we have attempted here to take advantage of this complexity by looking at pathway and looking at interactions between type 2 diabetes (T2D) and neurological diseases, due to their great clinical importance. T2D is known to affect neurological diseases but how it does this is generally unclear (though some vascular based mechanisms are usually considered) and it is very hard to study by hypothesis-driven biochical or endocrinological research. This is why we employed well-established bioinformatics methods and analytical approaches that examine functional disease overlaps in genes and pathways, and provide an important but agnostic tool to identify new factors that play a part in these comorbidity interactions and which, by implication, may be important pathogenic mechanisms for these and other related diseases. We studied the microarray gene expression datasets from publicly available repositories employing a network-based bioinformatics pipeline. We identified DEGs common to T2D and NDs and constructed diseasome networks to provide insights into the interactions of these comorbidities using the diesease-common DEGs. These DEGs enabled identification of associated dysregulated molecular pathways and related GO terms. One particular technical point is that a large number of pathways and GO categories were reduced by manual curation after filtering using a *p*-value threshold of 0.05. We identified different pathways by investigating cell proteins (i.e., gene products) and their interactions considering seven pathway databases.

In addition to the pathways and GO terms we investigated interactors of the products of our DEGs of interest using protein-protein interaction (PPI) analysis and hub protein identification as well as DEGs-TFs, and DEGs-miRNAs interaction that has not been previously studied for these diseases. These studies’ provide information about the molecules that could be the key drivers of pathogenesis for these comorbidities. The STRING database contains known protein-protein interactions which we used to identify the PPI for products of our genes of interest. For our purpose, we considered only experimentally verified PPI data, not predicted PPIs. We reconstructed the PPI based on the identified DEGs common to T2D and NDs and identified the central hub proteins using topological parameters. Among the hub proteins we identified, dynamin (DNM1) has been implicated in central nervous systems [81] and DNM2 is involved in Charcot-Marie-Tooth neuropathy [82]. However, a role for MYH14 in diabetes or neurodegenerative diseases is not reported. PACSIN2 expression has been found to be upregulated in diabetic kidney disease [83]. Borie et al studied the polymorphism of the TFRC gene involved in Parkinson’s disease [84]. Rahman et al. identified PDE4D commonly expressed in blood cell and brain tissues of AD [85]. A mutation of ENTPD1 has been identified in Spastic paraplegia type 64 in individuals diagnosed with suspected neurodegenerative disease patients [86]. To date, no role for PLK4, CDC20B, and CDC14A in NDs has been reported.

Transcription factors (TFs) are critical determinants of transcription of their various target genes, so their levels can identify potential biomarkers for neurodegenerative diseases. In this study, we identified relevant TFs as the regulator of the DEGs through TF-mRNA interaction networks that are relevant to the pathogenesis of T2D and NDs. TF-association has also been used by Rahman et al. in a network-based method to profile gene expression of DEGs associated with AD; they identified a number of AD-associated TFs, including JUN, YY1, E2F1, FOXC1, GATA2, SRF, USF2, PPARG, FOXL1, and NFIC [87], consistent with the present study. In contrast to TFs, microRNAs (∼22ntlong) act post-transcriptionally to regulate expression. These are single nucleotide RNA which bind target mRNA, leading to the target cleavage and reduced expression. These miRNAs have many advantages of non-invasive biomarkers and can be detected in body fluids such as urine, saliva and makes them potentially attractive as biomarkers. Indeed, there are miRNAs that show good potential as biomarkers for neurodegenerative diseases [88]. Thus, we studied DEGs-miRNA interaction networks to identify relevant miRNAs as potential targets for NDs. Among the identified miRNAs, the miRNA-335 was particularly associated with AD [71]. In addition, miRNA-16 was reported to be involved in apoptosis in neural cells [89]. Furthermore, the down-expression of this miRNA is involved in the accumulation of amyloid protein precursor (APP) protein in AD [90]. The miRNA-124 is found abundantly in neural cells and as known involvement in NDs. The reduced expression is miRNA associated with AD, PD, and HD [88]. There is no evidence for ND association with miR-17-5p although it has pathogenic actions both enhancing and suppressing tumour development depending on the cellular contexts [91].

The above indicates that our approach has the potential to reveal some of the important mechanisms that underlie disease pathogenesis and provide novel hypotheses of disease mechanisms and may identify new biomarkers. Such genetic data analyses will be a key element in the development of predictive medicine and elucidating the underlying mechanisms that connect T2D and NDs and may indicate possible new drug targets. Nevertheless, our data show some limitations. It should be noted that no clinical confirmation of the roles of proteins generated from our identified genes of interest. Furthermore, the low number of samples for some diseases analyzed which may not fully sample the disease-associated genes that we used to determine the common DEGs. Thus, further experimentation is needed to properly evaluate the biological significance of the identified potential targets candidates in this study.

## 5. Conclusions

The present study analyzed transcriptomics datasets of the T2D and neurodegenerative diseases employing a multi-omics approach to decode the overlapped genes that were expressed between T2D and NDs. The gene set enrichment analysis revealed significantly enriched dysregulated pathways. Integration of the overlapped DEGs with different biomolecular interaction networks yielded 10 hub proteins form protein-protein interaction, regulatory TFs from DEG-TF interactions analysis, and miRNAs from DEGs-miRNAs interactions analysis. All of these hub genes and pathways are novel, that is, they have not previously been shown to be important in these diseases or in the disease interactions and most of the TFs and miRNAs identified are also novel. In this way, the present study presented molecular signatures at proteins level (i.e., hub proteins and TFs), and RNA levels (i.e., mRNAs, miRNAs), pathway and GO level but further studies are needed to establish them as biomarkers. These results indicate differentially expressed genes of T2D that may be key to the progression of NDs and may give new insights into these diseases. It also points the way to identifying mechanistic links between the T2D and various ND and that explains why their association with T2D. This study also suggests that T2D shares several common multifactorial degenerative biological processes that contribute to neuronal death, which may, in turn, lead to functional impairment. Additionally, we believe that our high-throughput transcript analysis of tissues using rigorous agnostic approaches would allow the discovery of disease-modifying therapeutic targets. Treatments aimed at attenuating the identified dysregulated pathways have the potential to ameliorate neurological dysfunctions in the T2D patient. 

## Figures and Tables

**Table 1:** The differentially expressed gene (DEG) for all employed Datasets in the present study.

Disease Name	GEO Platform	Tissues/Cells	GEO Accession	Raw Genes	Case Samples	Control Samples	UP Reg. Genes	Down Reg. Genes
Type 2 Diabetes (T2D)	Affymetrix Human Genome U133 Plus 2.0 Array	Liver	GSE23343	54613	10	7	622	698
Alzheimer’s disease	Affymetrix Human Genome U133 Plus 2.0 Array	CA1 tissue	GSE28146	54675	22	8	847	759
Amyotrophic lateral sclerosis	Affymetrix Human Full Length HuGeneFL Array	Spinal cord	GSE833	22277	7	5	735	2166
Cerebral palsy	Affymetrix Human Genome U133 Plus 2.0 Array	Muscle	GSE31243	22277	20	20	243	345
Epilepsy disease	Affymetrix Human Genome U133 Plus 2.0 Array	Peripheral Blood	GSE22779	54675	12	4	882	1007
Huntington’s disease	Affymetrix Human Genome U133 Plus 2.0 Array	Whole Blood	GSE1751	22283	17	14	365	973
Multiple sclerosis	Affymetrix Human Genome U133 Plus 2.0 Array	Brain	GSE38010	33398	5	2	3987	3476
Parkinson’s disease	Affymetrix Human Genome U133A 2.0 Array	Brain	GSE19587	22277	12	10	1167	422

**Table 2:** Pathways common to T2D and the NDs revealed by the commonly expressed genes. These include significant pathways common to (**a**) T2D and AD (**b**) T2D and ALS (**c**) T2D and CP (**d**) T2D and ED (**e**) T2D and HD (**f**) T2D and MS and (**g**) T2D and PD.

(**a**) Common significant pathway common between T2D and AD		
**Pathway Name**	***p*** **-Value**	**Source**
Neuroregulin receptor degredation protein-1 Controls ErbB3 receptor recycling	3.78 $\times \;10^{-2}$	Biocarta
Cytokine-cytokine receptor interaction	1.17 $\times \;10^{-2}$	KEGG
Toll-like receptor signaling pathway	2.92 $\times \;10^{-2}$	KEGG
Glycosphingolipid biosynthesis	3.36 $\times \;10^{-2}$	KEGG
IL1-mediated signaling events	1.89 $\times \;10^{-2}$	NCI-Nature
TCR signaling in naive CD8+ T cells	4.53 $\times \;10^{-2}$	NCI-Nature
Ubiquitin proteasome pathway	3.09 $\times \;10^{-2}$	Panther
Ionotropic glutamate receptor pathway	3.36 $\times \;10^{-2}$	Panther
Glutamate Neurotransmitter Release Cycle	1.02 $\times \;10^{-2}$	Reactome
Signaling by Interleukins	1.32 $\times \;10^{-2}$	Reactome
Cytokine Signaling in the Immune system	1.83 $\times \;10^{-2}$	Reactome
Oxidative Stress-Induced Senescence	2.07 $\times \;10^{-2}$	Reactome
Neurotransmitter Release Cycle	4.23 $\times \;10^{-2}$	Reactome
Transmission across Chemical Synapses	4.68 $\times \;10^{-2}$	Reactome
Apoptosis Modulation and Signaling	2.97 $\times \;10^{-4}$	Wiki
(**b**) Common significant pathway common between T2D and ALS		
**Pathway Name**	***p*** **-Value**	**Source**
Neuroregulin receptor degredation protein-1 Controls ErbB3 receptor recycling	1.48 $\times \;10^{-3}$	Biocarta
Cytokine-cytokine receptor interaction	3.10 $\times \;10^{-3}$	KEGG
Glycosphingolipid biosynthesis	1.05 $\times \;10^{-2}$	KEGG
Ubiquitin mediated proteolysis	1.28 $\times \;10^{-2}$	KEGG
Glutamatergic synapse	2.85 $\times \;10^{-2}$	KEGG
Immune System	3.74 $\times \;10^{-3}$	Reactome
Innate Immune System	7.98 $\times \;10^{-3}$	Reactome
Insulin receptor signaling cascade	8.94 $\times \;10^{-3}$	Reactome
Cytokine Signaling in the Immune system	1.05 $\times \;10^{-2}$	Reactome
Neuronal System	1.17 $\times \;10^{-2}$	Reactome
Neurotransmitter Receptor Binding And Downstream Transmission in The Postsynaptic Cell	1.47 $\times \;10^{-2}$	Reactome
Transmission across Chemical Synapses	2.05 $\times \;10^{-2}$	Reactome
Adaptive Immune System	2.30 $\times \;10^{-2}$	Reactome
NO/cGMP/PKG mediated Neuroprotection	1.19 $\times \;10^{-2}$	Wiki
Toll-like Receptor Signaling	3.90 $\times \;10^{-2}$	Wiki
(**c**) Common significant pathway common between T2D and CP		
**Pathway Name**	***p*** **-Value**	**Source**
Focal adhesion	4.47 $\times \;10^{-3}$	KEGG
Dopaminergic synapse	9.70 $\times \;10^{-3}$	KEGG
Cell adhesion molecules (CAMs)	1.28 $\times \;10^{-2}$	KEGG
Rapid glucocorticoid signaling	2.65 $\times \;10^{-2}$	NCI-Nature
Inflammation mediated by chemokine and cytokine signaling pathway	4.19 $\times \;10^{-4}$	Panther
Adaptive Immune System	7.50 $\times \;10^{-6}$	Reactome
Immune System	1.47 $\times \;10^{-4}$	Reactome
Neurotransmitter Receptor Binding And Downstream Transmission In The Postsynaptic Cell	1.21 $\times \;10^{-2}$	Reactome
Electric Transmission Across Gap Junctions	1.66 $\times \;10^{-2}$	Reactome
Transmission across Electrical Synapses	1.66 $\times \;10^{-2}$	Reactome
Neuronal System	1.84 $\times \;10^{-2}$	Reactome
Neurofascin interactions	2.32 $\times \;10^{-2}$	Reactome
Transmission across Chemical Synapses	3.39 $\times \;10^{-2}$	Reactome
Inflammatory Response Pathway	4.53 $\times \;10^{-3}$	Wiki
Insulin signaling in human adipocytes	2.65 $\times \;10^{-2}$	Wiki
Toll-like Receptor Signaling Pathway	4.68 $\times \;10^{-2}$	Wiki
(**d**) Common significant pathway common between T2D and ED		
**Pathway Name**	***p*** **-Value**	**Source**
Cell adhesion molecules (CAMs)	1.14 $\times \;10^{-2}$	KEGG
Ubiquitin mediated proteolysis	4.95 $\times \;10^{-2}$	KEGG
TCR signaling in naive CD8+ T cells	4.03 $\times \;10^{-2}$	NCI-Nature
Rapid glucocorticoid signaling	4.70 $\times \;10^{-2}$	NCI-Nature
Apoptosis signaling pathway	2.35 $\times \;10^{-2}$	Panther
Ubiquitin proteasome pathway	2.75 $\times \;10^{-2}$	Panther
Adaptive Immune System	6.15 $\times \;10^{-3}$	Reactome
Oxidative Stress-Induced Senescence	1.75 $\times \;10^{-2}$	Reactome
Immune System	2.26 $\times \;10^{-2}$	Reactome
Deposition of new CENPA-containing nucleosomes at the centromere	3.90 $\times \;10^{-2}$	Reactome
Spinal Cord Injury	3.42 $\times \;10^{-2}$	Wiki
(**e**) Common significant pathway common between T2D and HD		
**Pathway Name**	***p*** **-Value**	**Source**
Neuroregulin receptor degredation protein-1 Controls ErbB3 receptor recycling	2.59 $\times \;10^{-4}$	Biocarta
Glycosphingolipid biosynthesis	1.54 $\times \;10^{-2}$	KEGG
Cell adhesion molecules (CAMs)	2.32 $\times \;10^{-2}$	KEGG
Cytokine-cytokine receptor interaction	3.53 $\times \;10^{-2}$	KEGG
Neurotrophic factor-mediated Trk receptor signaling	2.72 $\times \;10^{-2}$	NCI-Nature
Ubiquitin proteasome pathway	1.41 $\times \;10^{-2}$	Panther
Cholesterol biosynthesis	4.53 $\times \;10^{-2}$	Panther
Immune System	1.82 $\times \;10^{-3}$	Reactome
Cytokine Signaling in the Immune system	1.54 $\times \;10^{-2}$	Reactome
Innate Immune System	2.00 $\times \;10^{-2}$	Reactome
Adaptive Immune System	4.10 $\times \;10^{-2}$	Reactome
Toll-Like Receptors Cascades	2.12 $\times \;10^{-2}$	Reactome
NO/cGMP/PKG mediated Neuroprotection	1.67 $\times \;10^{-2}$	Wiki
Apoptosis	4.87 $\times \;10^{-2}$	Wiki
Tryptophan metabolism	1.60 $\times \;10^{-2}$	Wiki
(**f**) Common significant pathway common between T2D and MS		
**Pathway Name**	***p*** **-Value**	**Source**
Neurotrophin signaling pathway	1.17 $\times \;10^{-2}$	KEGG
Glycosphingolipid biosynthesis	1.36 $\times \;10^{-2}$	KEGG
Adipocytokine signaling pathway	1.39 $\times \;10^{-2}$	KEGG
Autophagy	1.69 $\times \;10^{-2}$	KEGG
Glutamatergic synapse	3.07 $\times \;10^{-2}$	KEGG
GABAergic synapse	3.68 $\times \;10^{-2}$	KEGG
Neurotrophic factor-mediated Trk receptor signaling	8.37 $\times \;10^{-3}$	NCI-Nature
Insulin/IGF pathway-protein kinase B signaling cascade	5.05 $\times \;10^{-3}$	Panther
Apoptosis signaling pathway	5.17 $\times \;10^{-3}$	Panther
Ubiquitin proteasome pathway	1.16 $\times \;10^{-2}$	Panther
Ionotropic glutamate receptor pathway	1.36 $\times \;10^{-2}$	Panther
Transmission across Chemical Synapses	8.34 $\times \;10^{-6}$	Reactome
Neuronal System	1.71 $\times \;10^{-5}$	Reactome
Neurotransmitter Receptor Binding And Downstream Transmission In The Postsynaptic Cell	1.77 $\times \;10^{-4}$	Reactome
Insulin receptor signaling cascade	2.65 $\times \;10^{-3}$	Reactome
Oxidative Stress-Induced Senescence	1.13 $\times \;10^{-2}$	Reactome
Brain-Derived Neurotrophic Factor (BDNF) signaling pathway	2.98 $\times \;10^{-2}$	Wiki
(**g**) Common significant pathway common between T2D and PD		
**Pathway Name**	***p*** **-Value**	**Source**
Neuroregulin receptor degredation protein-1 Controls ErbB3 receptor recycling	4.04 $\times \;10^{-4}$	Biocarta
Toll-like receptor signaling pathway	2.21 $\times \;10^{-3}$	KEGG
Cell adhesion molecules (CAMs)	7.20 $\times \;10^{-3}$	KEGG
Allograft rejection	1.70 $\times \;10^{-2}$	KEGG
Graft-versus-host disease	1.96 $\times \;10^{-2}$	KEGG
Intestinal immune network for IgA production	2.63 $\times \;10^{-2}$	KEGG
Innate Immune System	1.07 $\times \;10^{-3}$	Reactome
Insulin receptor signaling cascade	4.07 $\times \;10^{-3}$	Reactome
Immune System	1.50 $\times \;10^{-2}$	Reactome
Chemokine receptors bind chemokines	3.50 $\times \;10^{-2}$	Reactome
Adaptive Immune System	4.73 $\times \;10^{-2}$	Reactome
Transmission across Electrical Synapses	2.60 $\times \;10^{-2}$	Reactome

**Table 3:** Gene ontology identification of biological processes common to (**a**) T2D and AD (**b**) T2D and ALS (**c**) T2D and CP (**d**) T2D and ED (**e**) T2D and HD (**f**) T2D and MS and (**g**) T2D and PD.

(**a**) Common significant GOs of T2D and AD		
**GO ID**	**Pathway**	***p*** **-Value**
GO:0032000	positive regulation of fatty acid beta-oxidation	5.99 $\times \;10^{-3}$
GO:0016064	immunoglobulin mediated immune response	6.73 $\times \;10^{-3}$
GO:2000269	regulation of fibroblast apoptotic process	6.73 $\times \;10^{-3}$
GO:0031998	regulation of fatty acid beta-oxidation	8.22 $\times \;10^{-3}$
GO:0051588	regulation of neurotransmitter transport	1.05 $\times \;10^{-2}$
GO:0007498	mesoderm development	1.64 $\times \;10^{-2}$
GO:0051961	negative regulation of nervous system development	1.71 $\times \;10^{-2}$
GO:0046928	regulation of neurotransmitter secretion	2.08 $\times \;10^{-2}$
(**b**) Common significant GOs of T2D and ALS		
**GO ID**	**Pathway**	***p*** **-Value**
GO:0006352	DNA-templated transcription, initiation	2.46 $\times \;10^{-3}$
GO:0050870	positive regulation of T cell activation	4.48 $\times \;10^{-3}$
GO:0048935	peripheral nervous system neuron development	1.01 $\times \;10^{-2}$
GO:0021522	spinal cord motor neuron differentiation	1.01 $\times \;10^{-2}$
GO:0021559	trigeminal nerve development	1.01 $\times \;10^{-2}$
GO:2000145	regulation of cell motility	1.10 $\times \;10^{-2}$
GO:0048665	neuron fate specification	1.15 $\times \;10^{-2}$
GO:1902692	regulation of neuroblast proliferation	1.58 $\times \;10^{-2}$
GO:0048663	neuron fate commitment	2.01 $\times \;10^{-2}$
GO:2000177	regulation of neural precursor cell proliferation	2.15 $\times \;10^{-2}$
GO:0014033	neural crest cell differentiation	2.30 $\times \;10^{-2}$
GO:0045597	positive regulation of cell differentiation	3.23 $\times \;10^{-2}$
GO:0051961	negative regulation of nervous system development	3.28 $\times \;10^{-2}$
GO:0019228	neuronal action potential	3.42 $\times \;10^{-2}$
GO:0021953	central nervous system neuron differentiation	4.68 $\times \;10^{-2}$
GO:0050768	negative regulation of neurogenesis	4.82 $\times \;10^{-2}$
(**c**) Common significant GOs of T2D and CP		
**GO ID**	**Pathway**	***p*** **-Value**
GO:0071363	cellular response to growth factor stimulus	5.47 $\times \;10^{-3}$
GO:0048681	negative regulation of axon regeneration	6.38 $\times \;10^{-3}$
GO:0070571	negative regulation of neuron projection regeneration	7.18 $\times \;10^{-3}$
GO:0099590	neurotransmitter receptor internalization	8.77 $\times \;10^{-3}$
GO:0048679	regulation of axon regeneration	8.77 $\times \;10^{-3}$
GO:0021952	central nervous system projection neuron axonogenesis	8.77 $\times \;10^{-3}$
GO:0008045	motor neuron axon guidance	8.77 $\times \;10^{-3}$
GO:0106030	neuron projection fasciculation	8.77 $\times \;10^{-3}$
GO:0071880	adenylate cyclase-activating adrenergic receptor signaling pathway	1.67 $\times \;10^{-2}$
GO:0071875	adrenergic receptor signaling pathway	1.67 $\times \;10^{-2}$
GO:1990090	cellular response to nerve growth factor stimulus	1.82 $\times \;10^{-2}$
GO:0010977	negative regulation of neuron projection development	4.08 $\times \;10^{-2}$
GO:0016192	vesicle-mediated transport	4.18 $\times \;10^{-2}$
GO:0001934	positive regulation of protein phosphorylation	4.22 $\times \;10^{-2}$
(**d**) Common significant GOs of T2D and ED		
**GO ID**	**Pathway**	***p*** **-Value**
GO:0006897	endocytosis	6.45 $\times \;10^{-3}$
GO:0007599	hemostasis	1.15 $\times \;10^{-2}$
GO:0090286	cytoskeletal anchoring at the nuclear membrane	1.30 $\times \;10^{-2}$
GO:0034214	protein hexamerization	1.44 $\times \;10^{-2}$
GO:0034063	stress granule assembly	1.73 $\times \;10^{-2}$
GO:0097205	renal filtration	1.73 $\times \;10^{-2}$
GO:0035278	miRNA mediated inhibition of translation	1.73 $\times \;10^{-2}$
GO:0071470	cellular response to osmotic stress	2.15 $\times \;10^{-2}$
GO:0034656	nucleobase-containing small molecule catabolic process	2.30 $\times \;10^{-2}$
GO:0021953	central nervous system neuron differentiation	4.68 $\times \;10^{-2}$
(**e**) Common significant GOs of T2D and HD		
**GO ID**	**Pathway**	***p*** **-Value**
GO:0042127	regulation of cell proliferation	6.92 $\times \;10^{-5}$
GO:0022409	positive regulation of cell-cell adhesion	2.16 $\times \;10^{-4}$
GO:0048665	neuron fate specification	5.19 $\times \;10^{-3}$
GO:0048663	neuron fate commitment	9.06 $\times \;10^{-3}$
GO:0014033	neural crest cell differentiation	1.04 $\times \;10^{-2}$
GO:0043161	proteasome-mediated ubiquitin-dependent protein catabolic process	1.49 $\times \;10^{-2}$
GO:0007169	transmembrane receptor protein tyrosine kinase signaling pathway	2.65 $\times \;10^{-2}$
GO:0007399	nervous system development	3.43 $\times \;10^{-2}$
(**f**) Common significant GOs of T2D and MS		
**GO ID**	**Pathway**	***p*** **-Value**
GO:0070229	negative regulation of lymphocyte apoptotic process	4.32 $\times \;10^{-4}$
GO:0099590	neurotransmitter receptor internalization	5.27 $\times \;10^{-4}$
GO:0051588	regulation of neurotransmitter transport	8.67 $\times \;10^{-4}$
GO:0050804	modulation of chemical synaptic transmission	2.29 $\times \;10^{-3}$
GO:0046928	regulation of neurotransmitter secretion	3.50 $\times \;10^{-3}$
GO:2000146	negative regulation of cell motility	3.66 $\times \;10^{-3}$
GO:0090181	regulation of cholesterol metabolic process	7.75 $\times \;10^{-3}$
GO:0072657	protein localization to membrane	1.43 $\times \;10^{-2}$
GO:0007005	mitochondrion organization	1.60 $\times \;10^{-2}$
GO:0010595	positive regulation of endothelial cell migration	2.11 $\times \;10^{-2}$
GO:0010646	regulation of cell communication	2.17 $\times \;10^{-2}$
GO:0050658	RNA transport	2.70 $\times \;10^{-2}$
GO:0010628	positive regulation of gene expression	3.43 $\times \;10^{-2}$
GO:2000145	regulation of cell motility	4.71 $\times \;10^{-2}$
GO:0014033	neural crest cell differentiation	4.92 $\times \;10^{-2}$
(**g**) Common significant GOs of T2D and PD		
**GO ID**	**Pathway**	***p*** **-Value**
GO:0070486	leukocyte aggregation	9.16 $\times \;10^{-3}$
GO:0070584	mitochondrion morphogenesis	1.14 $\times \;10^{-2}$
GO:0090128	regulation of synapse maturation	1.26 $\times \;10^{-2}$
GO:0045624	positive regulation of T-helper cell differentiation	1.37 $\times \;10^{-2}$
GO:0048854	brain morphogenesis	1.37 $\times \;10^{-2}$
GO:0019228	neuronal action potential	2.73 $\times \;10^{-2}$
GO:0097061	dendritic spine organization	2.84 $\times \;10^{-2}$
GO:0030199	collagen fibril organization	3.40 $\times \;10^{-2}$
GO:0061448	connective tissue development	3.51 $\times \;10^{-2}$
GO:0048813	dendrite morphogenesis	3.73 $\times \;10^{-2}$
GO:0099601	regulation of neurotransmitter receptor activity	3.84 $\times \;10^{-2}$

**Table 4:** Summary of hub proteins identified by protein-protein interaction analysis encoded by DEGs that are common to type 2 diabetes (T2D) and neurological diseases (NDs).

Protein Symbol	Degree	Description	Feature
DNM1	65	Dynamin 1	GTP binding
DNM2	84	Dynamin 2	GTP binding and GTPase activity
MYH14	82	Myosin Heavy Chain 14	Calmodulin binding and motor activity
PACSIN2	60	Protein Kinase C And Casein Kinase Substrate In Neurons	Identical protein binding and lipid binding
TFRC	10	Transferrin Receptor	Double-stranded RNA binding
PDE4D	39	Phosphodiesterase 4D	Enzyme binding and protein domain specific binding
ENTPD1	32	Ectonucleoside Triphosphate Diphosphohydrolase 1	Hydrolase activity and nucleoside-diphosphatase activity
PLK4	45	Polo Like Kinase 4	Identical protein binding and protein kinase activity
CDC20B	20	Cell Division Cycle 20B	Pathways related to DNA damage response
CDC14A	36	Cell Division Cycle 14A	Phosphatase activity and phosphoprotein phosphatase activity

**Table 5:** Summary of transcriptional and/or post-transcriptional post regulatory biomolecules of differentially expressed genes overlapped between type 2 diabetes (T2D) and neurological diseases (NDs) that includes (**a**) Regulatory Transcription Factors and (**b**) Regulatory microRNAs.

(**a**) Regulatory Transcription Factors		
**Symbol**	**Description**	**Feature**
FOXC1	Forkhead Box C1	NA-binding transcription factor activity and transcription factor binding
GATA2	GATA binding protein 2	DNA-binding transcription factor activity and chromatin binding
FOXL1	Forkhead Box L1	NA-binding transcription factor activity and DNA-binding transcription factor activity, RNA polymerase II-specific
YY1	YY1 Transcription Factor	NA-binding transcription factor activity and transcription coactivator activity
E2F1	E2F transcription factor 1	DNA-binding transcription factor activity and transcription factor binding.
NFIC	Nuclear Factor I C	DNA-binding transcription factor activity and proximal promoter DNA-binding transcription activator activity, RNA polymerase II-specific
NFYA	Nuclear Transcription Factor Y Subunit Alpha	DNA-binding transcription factor activity and transcription regulatory region DNA binding
USF2	Upstream Transcription Factor 2, C-Fos Interacting	DNA-binding transcription factor activity and sequence-specific DNA binding
HINFP	Histone H4 Transcription Factor	DNA-binding transcription factor activity and enzyme binding
MEF2A	Myocyte Enhancer Factor 2A	DNA-binding transcription factor activity and protein heterodimerization activity
SRF	Serum Response Factor	DNA-binding transcription factor activity and sequence-specific DNA binding
NFKB1	Nuclear Factor Kappa B Subunit 1	DNA-binding transcription factor activity and sequence-specific DNA binding
PDE4D	Phosphodiesterase 4D	enzyme binding and protein domain specific binding
CREB1	CAMP Responsive Element Binding Protein 1	DNA-binding transcription factor activity and enzyme binding
SP1	Sp1 Transcription Factor	DNA-binding transcription factor activity and sequence-specific DNA binding
HOXA5	Homeobox A5	DNA-binding transcription factor activity and RNA polymerase II proximal promoter sequence-specific DNA binding
SREBF1	Sterol Regulatory Element Binding Transcription Factor 1	DNA-binding transcription factor activity and chromatin binding
TFAP2A	Transcription Factor AP-2 Alpha	DNA-binding transcription factor activity and sequence-specific DNA binding
STAT3	Signal Transducer And Activator Of Transcription 3	DNA-binding transcription factor activity and sequence-specific DNA binding
POU2F2	POU Class 2 Homeobox 2	DNA-binding transcription factor activity and protein domain specific binding
TP53	Tumor Protein P53	NA-binding transcription factor activity and protein heterodimerization activity
PPARG	Peroxisome Proliferator Activated Receptor Gamma	DNA-binding transcription factor activity and chromatin binding
JUN	Jun Proto-Oncogene, AP-1 Transcription Factor Subunit	sequence-specific DNA binding
(**b**) Regulatory microRNAs		
**Symbol**	**Description**	**Feature**
mir-335-5p	MicroRNA 335	Afflicted with Alzheimer’s disease
mir-16-5p	MicroRNA 16	Afflicted with apoptosis of neural cells
mir-93-5p	MicroRNA 93	Involved in DNA damage pathways
mir-17-5p	MicroRNA 17	Act as oncogene or tumour suppressor gene depending on the cellular context
mir-124-3p	MicroRNA 124	Abundant in the brain and involved in neurodegenerative disease

## References

[1] DeFronzo R.A., Ferrannini E., Groop L., Henry R.R., Herman W.H., Holst J.J., Hu F.B., Kahn C.R., Raz I., Shulman G.I. (2008). Type 2 diabetes mellitus. Nat. Rev. Dis. Primers.

[2] Mallorquí-Bagué N., Lozano-Madrid M., Toledo E., Corella D., Salas-Salvadó J., Cuenca-Royo A., Vioque J., Romaguera D., Martínez J.A., Wärnberg J. (2018). Type 2 diabetes and cognitive impairment in an older population with overweight or obesity and metabolic syndrome: Baseline cross-sectional analysis of the predimed-plus study. Sci. Rep..

[3] Xu L., Huang X., Ma J., Huang J., Fan Y., Li H., Qiu J., Zhang H., Huang W. (2017). Value of three-dimensional strain parameters for predicting left ventricular remodeling after ST-elevation myocardial infarction. Int. J. Cardiovasc. Imaging.

[4] American Diabetes Association (2004). Diagnosis and classification of diabetes mellitus. Diabetes Care.

[5] Xu L., Zhao H., Qiu J., Zhu W., Lei H., Cai Z., Lin W.H., Huang W., Zhang H., Zhang Y.T. (2015). The different effects of BMI and WC on organ damage in patients from a cardiac rehabilitation program after acute coronary syndrome. BioMed Res. Int..

[6] Mota M., Banini B.A., Cazanave S.C., Sanyal A.J. (2016). Molecular mechanisms of lipotoxicity and glucotoxicity in nonalcoholic fatty liver disease. Metabolism.

[7] Dolan C., Glynn R., Griffin S., Conroy C., Loftus C., Wiehe P.C., Healy M.L., Lawlor B. (2018). Brain complications of diabetes mellitus: A cross-sectional study of awareness among individuals with diabetes and the general population in Ireland. Diabet. Med..

[8] Mushtaq G., Khan J.A., Kumosani T.A., Kamal M.A. (2015). Alzheimer’s disease and type 2 diabetes via chronic inflammatory mechanisms. Saudi J. Biol. Sci..

[9] Verdile G., Fuller S.J., Martins R.N. (2015). The role of type 2 diabetes in neurodegeneration. Neurobiol. Dis..

[10] Bharadwaj P., Wijesekara N., Liyanapathirana M., Newsholme P., Ittner L., Fraser P., Verdile G. (2017). The link between type 2 diabetes and neurodegeneration: Roles for amyloid-*β*, amylin, and tau proteins. J. Alzheimer’s Dis..

[11] Porte D., Baskin D.G., Schwartz M.W. (2005). Insulin signaling in the central nervous system: A critical role in metabolic homeostasis and disease from *C. elegans* to humans. Diabetes.

[12] Morsi M., Kobeissy F., Magdeldin S., Maher A., Aboelmagd O., Johar D., Bernstein L. (2019). A shared comparison of diabetes mellitus and neurodegenerative disorders. J. Cell. Biochem..

[13] Chatterjee S., Mudher A. (2018). Alzheimer’s disease and type 2 diabetes: A critical assessment of the shared pathological traits. Front. Neurosci..

[14] Martinez-Valbuena I., Valenti-Azcarate R., Amat-Villegas I., Riverol M., Marcilla I., de Andrea C.E., Sánchez-Arias J.A., del Mar Carmona-Abellan M., Marti G., Erro M.E. (2019). Amylin as a potential link between type 2 diabetes and alzheimer disease. Ann. Neurol..

[15] D’Ovidio F., d’Errico A., Carnà P., Calvo A., Costa G., Chiò A. (2018). The role of pre-morbid diabetes on developing amyotrophic lateral sclerosis. Eur. J. Neurol..

[16] Crisham Janik M.D., Newman T.B., Cheng Y.W., Xing G., Gilbert W.M., Wu Y.W. (2013). Maternal diagnosis of obesity and risk of cerebral palsy in the child. J. Pediatr..

[17] Lu C.L., Chang Y.H., Sun Y., Li C.Y. (2018). A population-based study of epilepsy incidence in association with type 2 diabetes and severe hypoglycaemia. Diabetes Res. Clin. Pract..

[18] Montojo M.T., Aganzo M., González N. (2017). Huntington’s disease and diabetes: Chronological sequence of its association. J. Huntington’s Dis..

[19] Ruiz-Argüelles A., Méndez-Huerta M.A., Lozano C.D., Ruiz-Argüelles G.J. (2018). Metabolomic profile of insulin resistance in patients with multiple sclerosis is associated to the severity of the disease. Mult. Scler. Relat. Disord..

[20] Shaw K. (2019). Type 2 diabetes and Parkinson’s disease. Pract. Diabetes.

[21] Schmitz T.W., Nathan Spreng R., Alzheimer’s Disease Neuroimaging Initiative (2016). Basal forebrain degeneration precedes and predicts the cortical spread of Alzheimer’s pathology. Nat. Commun..

[22] Livingston G., Sommerlad A., Orgeta V., Costafreda S.G., Huntley J., Ames D., Ballard C., Banerjee S., Burns A., Cohen-Mansfield J. (2017). Dementia prevention, intervention, and care. Lancet.

[23] Al-Chalabi A., Hardiman O. (2013). The epidemiology of ALS: A conspiracy of genes, environment and time. Nat. Rev. Neurol..

[24] Dupuis L., Pradat P.F., Ludolph A.C., Loeffler J.P. (2011). Energy metabolism in amyotrophic lateral sclerosis. Lancet Neurol..

[25] Desport J.C., Torny F., Lacoste M., Preux P.M., Couratier P. (2005). Hypermetabolism in ALS: Correlations with clinical and paraclinical parameters. Neurodegener. Dis..

[26] Kioumourtzoglou M.A., Rotem R.S., Seals R.M., Gredal O., Hansen J., Weisskopf M.G. (2015). Diabetes mellitus, obesity, and diagnosis of amyotrophic lateral sclerosis: A population-based study. JAMA Neurol..

[27] Cerebral Palsy Guidance (2019). Cerebral Palsy and Diabetes. https://www.cerebralpalsyguidance.com/cerebral-palsy/associated-disorders/diabetes/.

[28] Schendel D.E., Schuchat A., Thorsen P. (2002). Public health issues related to infection in pregnancy and cerebral palsy. Ment. Retard. Dev. Disabil. Res. Rev..

[29] Marcovecchio M.L., Petrosino M.I., Chiarelli F. (2015). Diabetes and epilepsy in children and adolescents. Curr. Diabetes Rep..

[30] Soltesz G., Acsadi G. (1989). Association between diabetes, severe hypoglycemia, and electroencephalographic abnormalities. Arch. Dis. Child..

[31] Ferlazzo E., Gasparini S., Beghi E., Sueri C., Russo E., Leo A., Labate A., Gambardella A., Belcastro V., Striano P. (2016). Epilepsy in cerebrovascular diseases: Review of experimental and clinical data with meta-analysis of risk factors. Epilepsia.

[32] Schönberger S.J., Jezdic D., Faull R.L., Cooper G.J. (2013). Proteomic analysis of the human brain in Huntington’s Disease indicates pathogenesis by molecular processes linked to other neurodegenerative diseases and to type-2 diabetes. J. Huntington’s Dis..

[33] Lalić N.M., Marić J., Svetel M., Jotić A., Stefanova E., Lalić K., Dragašević N., Miličić T., Lukić L., Kostić V.S. (2008). Glucose homeostasis in Huntington disease: Abnormalities in insulin sensitivity and early-phase insulin secretion. Arch. Neurol..

[34] Hou W.H., Li C.Y., Chang H.H., Sun Y., Tsai C.C. (2017). A population-based cohort study suggests an increased risk of multiple sclerosis incidence in patients with type 2 diabetes mellitus. J. Epidemiol..

[35] Biosa A., Outeiro T.F., Bubacco L., Bisaglia M. (2018). Diabetes Mellitus as a Risk Factor for Parkinson’s Disease: A Molecular Point of View. Mol. Neurobiol..

[36] Green H., Tsitsi P., Markaki I., Aarsland D., Svenningsson P. (2019). Novel Treatment Opportunities Against Cognitive Impairment in Parkinson’s Disease with an Emphasis on Diabetes-Related Pathways. CNS Drugs.

[37] Barrett T., Wilhite S.E., Ledoux P., Evangelista C., Kim I.F., Tomashevsky M., Marshall K.A., Phillippy K.H., Sherman P.M., Holko M. (2012). NCBI geo: Archive for functional genomics data sets—Update. Nucleic Acids Res..

[38] Misu H., Takamura T., Takayama H., Hayashi H., Matsuzawa-Nagata N., Kurita S., Ishikura K., Ando H., Takeshita Y., Ota T. (2010). A liver-derived secretory protein, selenoprotein p, causes insulin resistance. Cell Metab..

[39] Blalock E.M., Buechel H.M., Popovic J., Geddes J.W., Landfield P.W. (2011). Microarray analyses of laser-captured hippocampus reveal distinct gray and white matter signatures associated with incipient alzheimer’s disease. J. Chem. Neuroanat..

[40] Dangond F., Hwang D., Camelo S., Pasinelli P., Frosch M.P., Stephanopoulos G., Stephanopoulos G., Brown R.H., Gullans S.R. (2004). Molecular signature of late-stage human als revealed by expression profiling of postmortem spinal cord gray matter. Physiol. Genom..

[41] Smith L.R., Chambers H.G., Subramaniam S., Lieber R.L. (2012). Transcriptional abnormalities of hamstring muscle contractures in children with cerebral palsy. PLoS ONE.

[42] Carlet M., Janjetovic K., Rainer J., Schmidt S., Panzer-Grümayer R., Mann G., Prelog M., Meister B., Ploner C., Kofler R. (2010). Expression, regulation and function of phosphofructo-kinase/fructose-biphosphatases (pfkfbs) in glucocorticoid-induced apoptosis of acute lymphoblastic leukemia cells. BMC Cancer.

[43] Borovecki F., Lovrecic L., Zhou J., Jeong H., Then F., Rosas H.D., Hersch S.M., Hogarth P., Bouzou B., Jensen R.V. (2005). Genome-wide expression profiling of human blood reveals biomarkers for huntington’s disease. Proc. Natl. Acad. Sci. USA.

[44] Han M.H., Lundgren D.H., Jaiswal S., Chao M., Graham K.L., Garris C.S., Axtell R.C., Ho P.P., Lock C.B., Woodard J.I. (2012). Janus-like opposing roles of cd47 in autoimmune brain inflammation in humans and mice. J. Exp. Med..

[45] Lewandowski N.M., Ju S., Verbitsky M., Ross B., Geddie M.L., Rockenstein E., Adame A., Muhammad A., Vonsattel J.P., Ringe D. (2010). Polyamine pathway contributes to the pathogenesis of parkinson disease. Proc. Natl. Acad. Sci. USA.

[46] Ritchie M.E., Phipson B., Wu D., Hu Y., Law C.W., Shi W., Smyth G.K. (2015). Limma powers differential expression analyses for rna-sequencing and microarray studies. Nucleic Acids Res..

[47] Shannon P., Markiel A., Ozier O., Baliga N.S., Wang J.T., Ramage D., Amin N., Schwikowski B., Ideker T. (2003). Cytoscape: A software environment for integrated models of biomolecular interaction networks. Genome Res..

[48] Moni M.A., Liò P. (2017). Genetic profiling and comorbidities of zika infection. J. Infect. Dis..

[49] Moni M.A., Liò P. (2014). comor: A software for disease comorbidity risk assessment. J. Clin. Bioinform..

[50] Moni M.A., Liò P. (2015). How to build personalized multi-omics comorbidity profiles. Front. Cell Dev. Biol..

[51] Kuleshov M.V., Jones M.R., Rouillard A.D., Fernandez N.F., Duan Q., Wang Z., Koplev S., Jenkins S.L., Jagodnik K.M., Lachmann A. (2016). Enrichr: A comprehensive gene set enrichment analysis web server 2016 update. Nucleic Acids Res..

[52] Jin L., Zuo X.-Y., Su W.-Y., Zhao X.-L., Yuan M.-Q., Han L.-Z., Zhao X., Chen Y.-D., Rao S.-Q. (2014). Pathway-based analysis tools for complex diseases: A review. Genom. Proteomics Bioinform..

[53] Gene Ontology Consortium (2014). Gene ontology consortium: Going forward. Nucleic Acids Res..

[54] Kanehisa M., Goto S., Sato Y., Furumichi M., Tanabe M. (2011). Kegg for integration and interpretation of large-scale molecular data sets. Nucleic Acids Res..

[55] Croft D., O’kelly G., Wu G., Haw R., Gillespie M., Matthews L., Caudy M., Garapati P., Gopinath G., Jassal B. (2010). Reactome: A database of reactions, pathways and biological processes. Nucleic Acids Res..

[56] Krupa S., Anthony K., Buchoff J.R., Day M., Hannay T., Schaefer C.F. (2007). The nci-nature pathway interaction database: A cell signaling resource. Nat. Preced..

[57] Slenter D.N., Kutmon M., Hanspers K., Riutta A., Windsor J., Nunes N., Mélius J., Cirillo E., Coort S.L., Digles D. (2017). Wikipathways: A multifaceted pathway database bridging metabolomics to other omics research. Nucleic Acids Res..

[58] BioCarta N.D. (2001). Biotech Software & Internet Report. RG J..

[59] Mi H., Thomas P. (2009). PANTHER pathway: An ontology-based pathway database coupled with data analysis tools. Methods Mol. Biol..

[60] Trupp M., Altman T., Fulcher C.A., Caspi R., Krummenacker M., Paley S., Karp P.D. (2010). Beyond the genome (BTG) is a (PGDB) pathway genome database: HumanCyc. Genome Biol..

[61] De Las Rivas J., Fontanillo C. (2010). Protein–protein interactions essentials: Key concepts to building and analyzing interactome networks. PLoS Comput. Biol..

[62] Szklarczyk D., Franceschini A., Wyder S., Forslund K., Heller D., Huerta-Cepas J., Simonovic M., Roth A., Santos A., Tsafou K.P. (2014). String v10: Protein–protein interaction networks, integrated over the tree of life. Nucleic Acids Res..

[63] Khan A., Fornes O., Stigliani A., Gheorghe M., Castro-Mondragon J.A., van der Lee R., Bessy A., Cheneby J., Kulkarni S.R., Tan G. (2017). Jaspar 2018: Update of the open-access database of transcription factor binding profiles and its web framework. Nucleic Acids Res..

[64] Sethupathy P., Corda B., Hatzigeorgiou A.G. (2006). Tarbase: A comprehensive database of experimentally supported animal microrna targets. RNA.

[65] Hsu S.-D., Lin F.-M., Wu W.-Y., Liang C., Huang W.-C., Chan W.-L., Tsai W.-T., Chen G.-Z., Lee C.-J., Chiu C.M. (2010). mirtarbase: A database curates experimentally validated microrna–target interactions. Nucleic Acids Res..

[66] Xia J., Gill E.E., Hancock R.E.W. (2015). Networkanalyst for statistical, visual and network-based meta-analysis of gene expression data. Nat. Protoc..

[67] Davis S., Meltzer P.S. (2007). GEOquery: A bridge between the Gene Expression Omnibus (GEO) and BioConductor. Bioinformatics.

[68] Gentleman R., Carey V., Huber W., Hahne F. (2015). Genefilter: Methods for Filtering Genes from High-Throughput Experiments. https://bioconductor.riken.jp/packages/3.0/bioc/html/genefilter.html.

[69] Chen S.-H., Chin C.-H., Wu H.-H., Ho C.-W., Ko M.-T., Lin C.-Y. cyto-hubba: A cytoscape plug-in for hub object analysis in network biology. Proceedings of the 20th International Conference on Genome Informatics.

[70] Cheng C., Alexander R., Min R., Leng J., Yip K.Y., Rozowsky J., Yan K.K., Dong X., Djebali S., Ruan Y. (2012). Understanding transcriptional regulation by integrative analysis of transcription factor binding data. Genome Res..

[71] Moradifard S., Hoseinbeyki M., Ganji S.M., Minuchehr Z. (2018). Analysis of microRNA and gene expression profiles in Alzheimer’s disease: A meta-analysis approach. Sci. Rep..

[72] Van Cauwenberghe C., Van Broeckhoven C., Sleegers K. (2016). The genetic landscape of Alzheimer disease: Clinical implications and perspectives. Genet. Med..

[73] Anjana M., Ahuja Y.R. (2010). Genes associated with Alzheimer Disease. Neurol. Asia.

[74] Eykens C., Robberecht W. (2015). The Genetic basis of amyotrophic lateral sclerosis: Recent breakthroughs. Adv. Genom. Genet..

[75] Fahey M.C., Maclennan A.H., Kretzschmar D., Gecz J., Kruer M.C. (2017). The genetic basis of cerebral palsy. Dev. Med. Child Neurol..

[76] GeneDx Genetic Testing for Epilepsy: A Guide for Patients. https://www.genedx.com/wpcontent/uploads/crm_docs/91040_Epilepsy-Patient-Guide.pdf.

[77] Myers C.T., Mefford H.C. (2015). Advancing epilepsy genetics in the genomic era. Genome Med..

[78] Arning L., Epplen J.T. (2012). Genetic modifiers of Huntington’s disease: Beyond CAG. Future Neurol..

[79] Baranzini S.E. (2011). Revealing the genetic basis of multiple sclerosis: Are we there yet?. Curr. Opin. Genet. Dev..

[80] Redenšek S., Trošt M., Dolžan V. (2017). Genetic determinants of Parkinson’s disease: Can they help to stratify the patients based on the underlying molecular defect?. Front. Aging Neurosci..

[81] Antoni Romeu and Lluís Arola (2014). Classical dynamin dnm1 and dnm3 genes attain maximum expression in the normal human central nervous system. BMC Res. Notes.

[82] Sidiropoulos P.N.M., Miehe M., Bock T., Tinelli E., Oertli C.I., Kuner R., Meijer D., Wollscheid B., Niemann A., Suter U. (2012). Dynamin 2 mutations in charcot–marie–tooth neuropathy highlight the importance of clathrin-mediated endocytosis in myelination. Brain.

[83] Dumont V., Tolvanen T.A., Kuusela S., Wang H., Nyman T.A., Lindfors S., Tienari J., Nisen H., Suetsugu S., Plomann M. (2017). Pacsin2 accelerates nephrin trafficking and is up-regulated in diabetic kidney disease. FASEB J..

[84] Borie C., Gasparini F., Verpillat P., Bonnet A.-M., Agid Y., Hetet G., Brice A., Dürr A., Grandchamp B., French Parkinson’s Disease Genetic Study Group (2002). Association study between iron-related genes polymorphisms and parkinson’s disease. J. Neurol..

[85] Rahman M., Islam T., Shahjaman M., Zaman T., Faruquee H.M., Jamal M.A.H.M., Huq F., Quinn J.M.W., Moni M.A. (2019). Discovering biomarkers and pathways shared by alzheimer’s disease and ischemic stroke to identify novel therapeutic targets. Medicina.

[86] Mamelona J., Crapoulet N., Marrero A. (2019). A new case of spastic paraplegia type 64 due to a missense mutation in the entpd1 gene. Hum. Genome Var..

[87] Rahman M.R., Islam T., Turanli B., Zaman T., Faruquee H.M., Rahman M.M., Mollah M.N.H., Nanda R.K., Arga K.Y., Gov E. (2019). Network-based approach to identify molecular signatures and therapeutic agents in alzheimer’s disease. Comput. Biol. Chem..

[88] Godlewski J., Lenart J., Salinska E. (2019). Microrna in brain pathology: Neurodegeneration the other side of the brain cancer. Non-Coding RNA.

[89] Persengiev S.P., Kondova I.I., Bontrop R.E. (2012). The impact of micrornas on brain aging and neurodegeneration. Curr. Gerontol. Geriatr. Res..

[90] Liu W., Liu C., Zhu J., Shu P., Yin B., Gong Y., Qiang B., Yuan J., Peng X. (2012). Microrna-16 targets amyloid precursor protein to potentially modulate alzheimer’s-associated pathogenesis in samp8 mice. Neurobiol. Aging.

[91] Cloonan N., Brown M.K., Steptoe A.L., Wani S., Chan W.L., Forrest A.R.R., Kolle G., Gabrielli B., Grimmond S.M. (2008). The mir-17-5p microrna is a key regulator of the g1/s phase cell cycle transition. Genome Biol..

